# Acute hydrocephalus caused by intraspinal neurocysticercosis: case report

**DOI:** 10.1186/1756-0500-7-2

**Published:** 2014-01-02

**Authors:** Seok-Won Kim, Hui Sun Wang, Chang Il Ju, Dong-Min Kim

**Affiliations:** 1 Department of Neurosurgery, Chosun University College of Medicine, Gwang-ju, South Korea; 2 Department of Internal Medicine, Chosun University College of Medicine, 588 Seosuk-dong, Dong-gu, Gwangju 501-717, South Korea

**Keywords:** Spinal, Neurocysticercosis, Hydrocephalus

## Abstract

**Background:**

Intraspinal neurocysticercosis is an uncommon manifestation that may present as an isolated lesion. Furthermore, acute hydrocephalus caused by isolated intraspinal neurocysticercosis without concomitant cerebral involvement is extremely rare.

**Case presentation:**

A 64-year-old man presented with a history of severe headache, an unsteady gait, and occasional urinary incontinence. Magnetic resonance imaging of the thoraco-lumbar spine revealed multiple, cystic, contrast-enhancing intraspinal lesions. A computed tomographic scan of the brain showed marked ventricular dilatation but no intraparenchymal lesions or intraventricular cysticercal lesions. This case of acute hydrocephalus was found to be caused by isolated intraspinal neurocysticercosis and was treated by ventriculoperitoneal shunt placement and surgical removal of the intraspinal lesions (which were histologically confirmed as neurocysticercosis), followed by administration of dexamethasone and albendazole.

**Conclusion:**

Isolated spinal neurocysticercosis should be considered in the differential diagnosis of acute hydrocephalus when no explanation is found in the brain, particularly in geographical regions endemic for cysticercosis.

## Background

Cysticercosis is a common parasitic disease that invades the central nervous system; in humans, this condition is caused by encysted larvae of *Taenia solium*. Neurocysticercosis (NCC) of the central nervous system is classified by location as parenchymal, subarachnoid, intraventricular, or spinal. The spinal type is rare and accounts for only 1.0-5.8% of cases, and isolated spinal NCC without intracranial involvement accounts for ~25% of these cases [1-3]. Furthermore, acute hydrocephalus due to isolated spinal NCC is extremely rare because most patients with spinal NCC also present evidence of cerebral disease [4].

Here, we report a rare case of isolated intraspinal NCC exhibiting clinical features of acute hydrocephalus. In addition, we discuss the principles of the diagnosis and treatment of intraspinal NCC and provide a review of the literature.

## Case presentation

**Figure 1:**
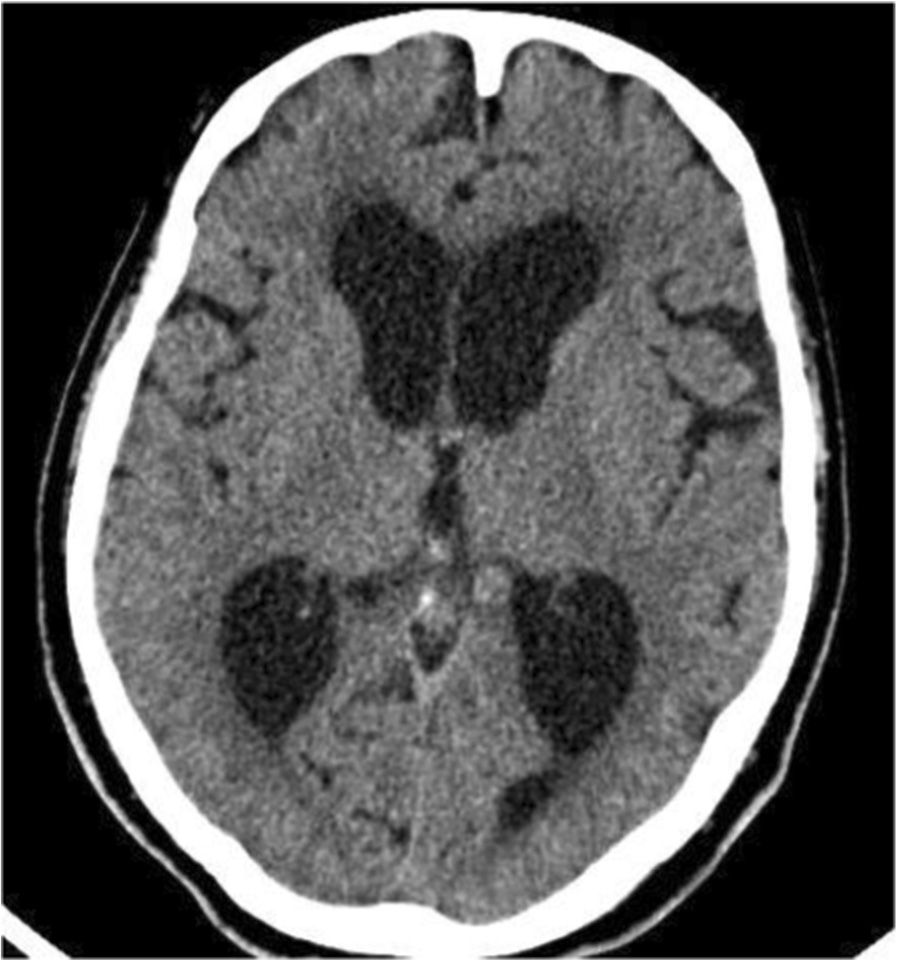
Brain computed tomography scan showing marked ventricular dilatation without calcification.

**Figure 2:**
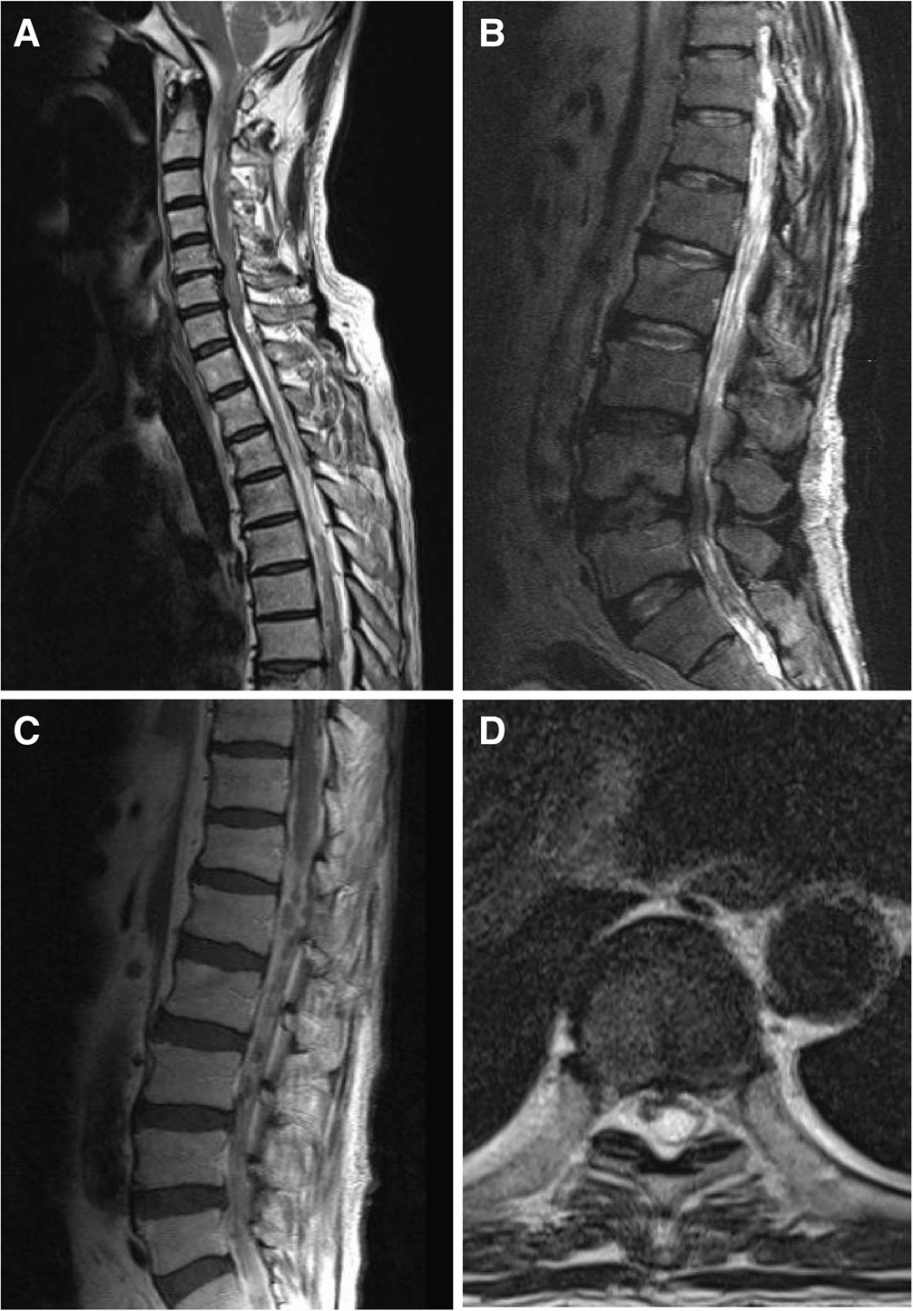
**Whole-spine magnetic resonance images of the patient. A**, **B**, **C**, **D**: Sagittal and axial T2-weighted images and postcontrast T1-weighted image showing multiple diffuse abnormal signals.

A 64-year-old man was admitted to our institute with a history of severe headache, an unsteady gait, and occasional urinary incontinence. These symptoms began 2 days prior to admission, and the patient had previously been healthy without medical concerns. He denied a previous history of consuming raw pork meat. On examination, the patient was alert but disoriented with respect to time and place. No significant pathological reflex was detected, and papilledema was absent. His lower extremities were hypotonic, and the strength of both legs, particularly in the proximal musculature, was diminished. A computed tomographic (CT) scan of the brain showed marked ventricular dilatation but no intraparenchymal lesions or intraventricular cysticercal lesions (Figure 1). In addition, no subcutaneous nodules were found. The peripheral blood findings revealed a leukocyte count of 8700/mm^3^ (eosinophils 3%) and a hemoglobin level of 12.8 mg/dL, although the hematological findings were unremarkable. Motor examination showed normal strength in both upper extremities but reduced strength in both lower extremities (Grade 4/5 on the Medical Research Council [MRC] scale). Under a diagnosis of acute hydrocephalus, lumbar tapping was attempted repeatedly, but cerebrospinal fluid (CSF) was not obtained despite adequate lumbar puncture. Magnetic resonance imaging (MRI) of the thoraco-lumbar spine revealed multiple cystic contrast-enhancing intraspinal lesions extending from C7 to S1 (Figure 2). Ventriculoperitoneal shunting and microsurgical removal of the lesions following laminectomy from T12 to L1 and L3 to L4 were performed under general anesthesia. During surgery, lesions of a mixed solid and cystic nature were detected. Subsequent pathological findings showed fibrosis, local calcification, and chronic inflammation associated with the cysticercosis cyst walls.

**Figure 3:**
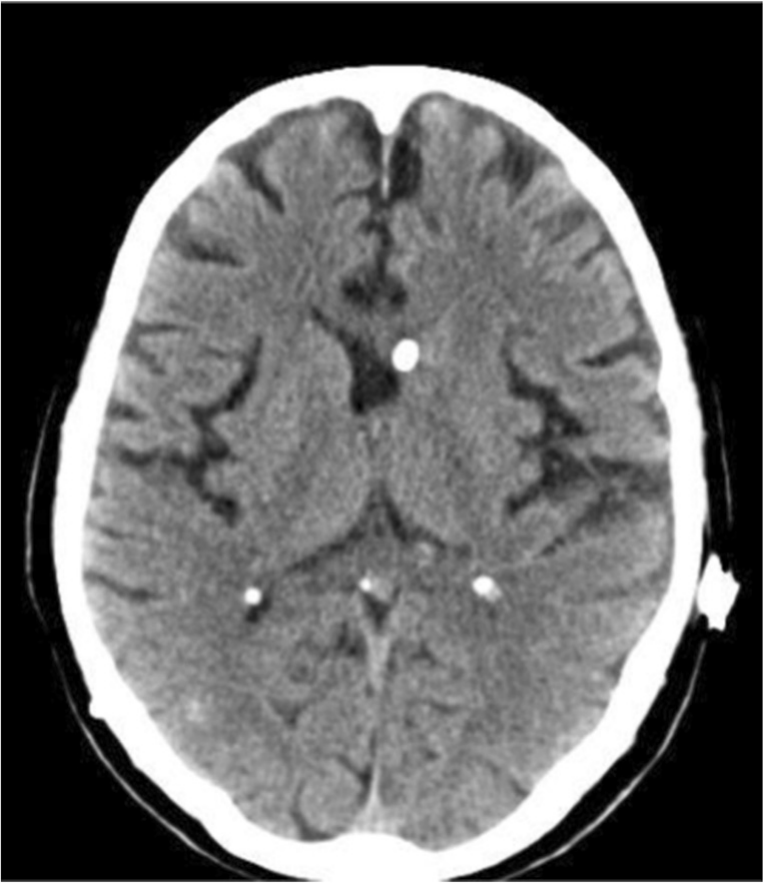
Brain computed tomography scan showing improved ventricular dilatation after ventriculoperitoneal shunting.

Postoperatively, an anticysticercal agent (Albendazole, Zentel® 400 mg, manufactured by Smith Kline Beecham Pharmaceuticals) and an oral steroid were administered for 6 weeks. During the early postoperative period, the patient remained awake, alert, and oriented, although his muscle strength and occasional urinary incontinence were not improved. After the 6-month follow-up period, these symptoms remained, and the patient was unable to ambulate without assistance despite improvements in ventricular dilation (Figure 3).

## Discussion

NCC is the most common parasitic disease capable of invading the human central nervous system and is caused by encysted larvae of *T. solium*. NCC typically involves the brain parenchyma, intracranial subarachnoid space, or ventricular system and can cause ventricular dilatation. The disease is often self-limiting unless hydrocephalus requires surgical intervention [5]. Intraspinal involvement in cases of cysticercosis is rare even in endemic regions and is encountered in only 1.0-5.8% of patients with NCC. Furthermore, in most of these cases, intraspinal involvement is associated with concomitant cerebral involvement. Accordingly, isolated spinal involvement in cases of cysticercosis of an intramedullary or extramedullary nature is extremely rare [5-7]. Callaconde *et al.*[8] reported that asymptomatic spinal subarachnoid disease is commonly observed in patients with basal subarachnoid NCC (61%). Therefore, subarachnoid disease of the spine is likely underrecognized and underreported [8].

Sotelo *et al.*[9] reported a frequency of spinal-type NCC of only 0.77% among 753 cases of active neurocysticercosis; this form may require more aggressive treatment due to the natural confines of the spinal canal. Furthermore, the location and size of the lesions, the inflammatory response, and arachnoid scarring generated by cyst breakdown are important causes of symptoms [10]. The frequency of intraspinal NCC is likely significantly lower than that of cerebral parenchymal NCC due to a smaller vessel diameter, low blood flow, and the relative hardness of the spinal cord compared to the cerebrum [11]. Acute hydrocephalus accompanying elevated intracranial pressure can be caused by multiple cysticerci within the spinal canal and by total obstruction of the subarachnoid natural CSF pathway without concomitant cerebral involvement. In addition, the parasite can reach the spinal cord parenchyma or CSF via several routes. These routes include the hemopoietic venous route via retrograde blood flow through the intervertebral venous plexus and intervertebral veins. In the ventriculoependymal route, intraventricular hypertension promotes ependymal canal dilation, allowing cysticerci to migrate from the fourth ventricle into the spinal cord. The subarachnoid route enables transpinal migration into the spinal cord and could explain the intramedullary form of this condition. Finally, although unlikely, continuity from the intestinal mucosae to the intradural space may allow parasites to pass throughout several organic tissue layers without utilizing the blood stream [5]. Treatment can be divided into 2 types: curative treatment and the prevention of re-infestation and/or dissemination of the parasitosis. Furthermore, elimination of the direct causes of neurological deficits (spinal cord compression, inflammation, or intracranial hypertension), restoration of iatrogenic CSF pathway obstruction, anti-inflammatory drug therapy, and anticysticidal drug treatment should be considered and treated promptly. In the case described here, we performed open decompressive surgery to excise multiple cysticerci and initiated CSF drainage to relieve the symptoms of hydrocephalus. In addition, corticosteroid agents were used to control the inflammatory reactions against cysticerci and cysticercal toxins, such as those released during episodes of cysticercal meningitis. However, the outcome of this case of predominant spinal NCC was poor despite surgical and medical intervention.

In this case, brain imaging analysis showed symmetric dilatation of all 4 ventricles without any obvious cause in the brain. Therefore, the pathophysiology of hydrocephalus may have been secondary to chronic arachnoiditis due to cysticercosis [12]. However, a major limitation of this study was that we could not evaluate the patient’s brain MRI due to his refusal. A CT scan of the brain cannot completely rule out subarachnoid disease of the basilar cisterns or Sylvian fissure, and we could therefore not rule out the possibility that his clinical presentation of headache with ventriculomegaly on brain CT was consistent with chronic inflammation in the subarachnoid CSF space extending to the intracranium. Although intraspinal NCC is rare even in endemic regions, it should be considered as a possible cause of acute hydrocephalus.

## Conclusion

In conclusion, isolated spinal NCC should be considered in the differential diagnosis of acute hydrocephalus when no explanation is found in the brain, particularly in regions endemic for cysticercosis.

## Consent

Written informed consent was obtained from the patient for publication of this Case Report and any accompanying images. A copy of the written consent is available for review by the Editor-in-Chief of this journal.

## Abbreviations

NCC: Neurocysticercosis; CT: Computed tomographic scan; MRI: Magnetic resonance imaging; CSF: Cerebrospinal fluid.

## Competing interests

The authors declare that they have no competing interests.

## Authors’ contributions

SK and HW performed the diagnosis and surgery. SK and CJ drafted the manuscript and revised it critically for important intellectual content. CJ and DK made substantial contributions to the study conception and design. SK and DK granted final approval of the version to be published. All authors read and approved the final manuscript.
